# Recent and historical recombination in the admixed Norwegian Red cattle breed

**DOI:** 10.1186/1471-2164-12-33

**Published:** 2011-01-14

**Authors:** Marte Sodeland, Matthew Kent, Ben J Hayes, Harald Grove, Sigbjørn Lien

**Affiliations:** 1 Department of Animal and Aquacultural Sciences, Centre for Integrative Genetics, Norwegian University of Life Sciences, N-1432 Aas, Norway; 2 Biosciences Research Division, Department of Primary Industries Victoria, Melbourne, 3083, Australia

## Abstract

**Background:**

Comparison of recent patterns of recombination derived from linkage maps to historical patterns of recombination from linkage disequilibrium (LD) could help identify genomic regions affected by strong artificial selection, appearing as reduced recent recombination. Norwegian Red cattle (NRF) make an interesting case study for investigating these patterns as it is an admixed breed with an extensively recorded pedigree. NRF have been under strong artificial selection for traits such as milk and meat production, fertility and health.

While measures of LD is also crucial for determining the number of markers required for association mapping studies, estimates of recombination rate can be used to assess quality of genomic assemblies.

**Results:**

A dataset containing more than 17,000 genome-wide distributed SNPs and 2600 animals was used to assess recombination rates and LD in NRF. Although low LD measured by r^2 ^was observed in NRF relative to some of the breeds from which this breed originates, reports from breeds other than those assessed in this study have described more rapid decline in r^2 ^at short distances than what was found in NRF. Rate of decline in r^2 ^for NRF suggested that to obtain an expected r^2 ^between markers and a causal polymorphism of at least 0.5 for genome-wide association studies, approximately one SNP every 15 kb or a total of 200,000 SNPs would be required. For well known quantitative trait loci (QTLs) for milk production traits on *Bos Taurus *chromosomes 1, 6 and 20, map length based on historic recombination was greater than map length based on recent recombination in NRF.

Further, positions for 130 previously unpositioned contigs from assembly of the bovine genome sequence (Btau_4.0) found using comparative sequence analysis were validated by linkage analysis, and 28% of these positions corresponded to extreme values of population recombination rate.

**Conclusion:**

While LD is reduced in NRF compared to some of the breeds from which this admixed breed originated, it is elevated over short distances compared to some other cattle breeds. Genomic regions in NRF where map length based on historic recombination was greater than map length based on recent recombination coincided with some well known QTL regions for milk production traits.

Linkage analysis in combination with comparative sequence analysis and detection of regions with extreme values of population recombination rate proved to be valuable for detecting problematic regions in the Btau_4.0 genome assembly.

## Background

The historical pattern of recombination in the population of genomes of a species or breed contain an enormous amount of information on history of population size, including expansions and contractions, gene flow between other breeds, and selection [1]. It has also been demonstrated that rate of recombination is not uniform across a chromosomal segment, rather recombination events tend to occur in recombination hotspots [2,3]. The pattern of linkage disequilibrium (LD) in the current generation of a species reflects all of these processes. While the pattern of LD therefore contains much information, deciphering the relative contribution of each process to the current pattern of LD is challenging [1,4-11].

Some additional insight into the relative contribution of each process can be gained from comparing historical patterns of recombination inferred from LD to recent patterns of recombination inferred from genetic maps. One hypothesis would be that in genome regions where large discrepancies are observed between map distances inferred from LD and genetic map distances, strong selection is occurring. Norwegian Red cattle (NRF) was developed mainly through crosses of old Norwegian breeds with other Scandinavian breeds like Swedish Red and White, Black and White Swedish and Finnish Ayrshire. Pedigree data has been recorded since formation of NRF, and the breed has been under strong artificial selection for traits such as milk and meat production, fertility and health. A further attraction of using NRF for this type of study is the extensive pedigree data available, assisting determination of frequency of recombination events between adjacent markers. The extent of LD in cattle has been investigated in a number of studies [7,12-15]. Relative to humans cattle display elevated LD, which is likely due to small recent effective population size generally observed in livestock populations [7,12-16]. Previous studies have shown some variation between cattle breeds in rate of decline in LD with increasing distance between genetic markers [15,17,18], which is also at least partly attributable to population history.

Another application for recombination rate estimates is within validation of positioning and assembly of genome contigs by linkage analysis. The bovine genome has recently been sequenced by a combined bacterial artificial chromosome and whole-genome shotgun approach [19]. The resulting Btau_4.0 assembly has contig and scaffold N50 sizes of 48.7 kb and 1.9 Mb respectively, and represents 95% of the total genome sequence placed on the 29 autosomes and the X chromosome. Construction of genetic maps in NRF was used to assess quality of the Btau_4.0 assembly and indicated a positional error rate of less than 0.8% [19].

The sequencing and assembly of larger genomes is a complex task with many challenges, and will usually result in imperfect assemblies. The desire to build a complete assembly is often at odds with the application of stringent merging criteria, and a compromise strategy resulting in longer scaffolds containing some assembly errors is usually the end result [20-22].

Aim of this study was to provide maps of historic and recent recombination rate in NRF, and then to attempt to use these to infer aspects of population history. Recombination rate information was also used to assess quality of the Btau_4.0 assembly.

## Results and discussion

**Figure 1:**
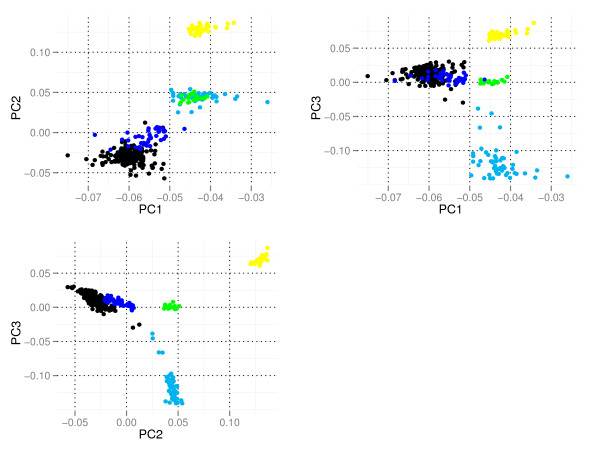
**Principal component analysis**. Principal component (PC) analysis of genomic relationships among Norwegian Red cattle (black), Holsteins (light blue), Sided Troender and Nordland Cattle (green), Finnish Ayrshires (dark blue), and Icelandic Cattle (yellow). Plots are PC2 versus PC1, PC3 versus PC1 and PC3 versus PC2.

A total of 2,480 paternal half-sib NRF sires and 109 founding NRF sires were genotyped using the Affymetrix 25 K MIP array. The final male genetic map contained 17,347 SNPs distributed on the 29 *Bos Taurus *chromosomes (BTAs) [19], and distributions of spacing between adjacent SNPs and minor-allele frequency (MAF) for the SNPs are presented in additional files 1 and 2. In order to examine the relationships between NRF and cattle breeds that have contributed to the development of NRF, 53 Holstein, 40 Finnish Ayrshire, 19 Sided Troender and Nordland Cattle and 39 Icelandic bulls were also genotyped. Icelandic cattle are believed to have been derived from old Norwegian breeds approximately 1000 years ago. Genetic distances between breeds were investigated using a principal component analysis of the genomic relationship matrix among individuals of different and the same breed [23]. Principal component 1 (PC1), PC2 and PC3 are plotted in Figure 1. For PC1 and PC2, the Finnish Ayrshires and NRF animals group together, likely reflecting the high level of contribution of Finnish Ayrshire to NRF. Icelandic cattle appear genetically distinct, perhaps reflecting the 1000 years of genetic isolation of this breed from the other breeds. PC3 separates Holsteins from the other breeds. The principal component analysis also clearly demonstrates heterogeneity in composition among NRF. For example, some NRF animals have higher than average levels of relationship to Finnish Ayrshires, while other NRF animals have high levels of relationship with Holsteins.

**Figure 2:**
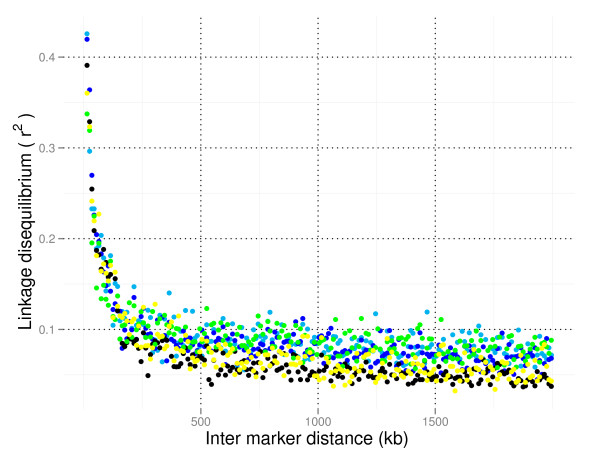
**Extent of linkage disequilibrium**. Extent of linkage disequilibrium (r^2^) in Norwegian Red cattle (black), Holsteins (light blue), Sided Troender and Nordland Cattle (green), Finnish Ayrshires (dark blue), and Icelandic Cattle (yellow).

The extent of LD in each breed was assessed by average r^2 ^for pairs of markers binned by distance between them (Figure 2). At short distances (<100 kb) Icelandic cattle had highest LD, likely reflecting small effective population size. NRF had lower levels of LD at comparable distances, especially distance greater than 100 kb, than any of the other breeds. The low levels of LD observed in NRF relative to the other breeds is likely due to elevated heterogeneity in NRF from historic admixture, recent attempts to control inbreeding and gene flow through import of sires from other Nordic countries [24].

For NRF the highest and lowest chromosomal mean values for r^2 ^were found on BTA22 and BTA1, and highest and lowest mean values of r^2 ^for inter marker distances less than 10 Mb were found for BTA5 and BTA19. Chromosomal mean values for NRF for r^2^, and for r^2 ^for inter marker distances less than 10 Mb, for all chromosomes are presented in Additional file 3.

At very short inter-marker distances, the level of LD in NRF was high (Table 1). A mean r^2 ^of 0.5 or more was observed for SNPs positioned less than 10 kb apart while a mean r^2 ^of 0.3 or more was observed for SNPs positioned less than 30 kb apart. The results suggest that to obtain an expected r^2 ^between markers and a causal polymorphism of at least 0.5 for genome-wide association studies, approximately one SNP every 15 kb or a total of 200,000 SNPs would be required for the 2.87 Gb genome. A report of decline in r^2 ^with increasing distance between SNPs in Australian Holstein-Friesian cattle [15] describes quite similar results as for NRF at these short distances. Reports from other breeds have described similar or more rapid decline in r^2 ^at short distances than what was found in NRF [17,18]. However, long range LD (Figure 2) is lower in NRF than in these other breeds.

**Table 1:** Expected linkage disequilibrium by inter-marker distance

Distance (kb)	r^2 ^mean	r^2 ^sd
0-1	0.7497	0.34175332
1-5	0.5960	0.35871376
5-10	0.4770	0.36559827
10-20	0.3524	0.37987201
20-30	0.2680	0.39594648
30-40	0.2211	0.40066317
40-50	0.2187	0.3976346
50-100	0.1543	0.36471807

Whole-genome mean r^2 ^for bins of short inter-marker distances (0 to 100 kb) between syntenic SNPs in Norwegian Red cattle.

**Figure 3:**
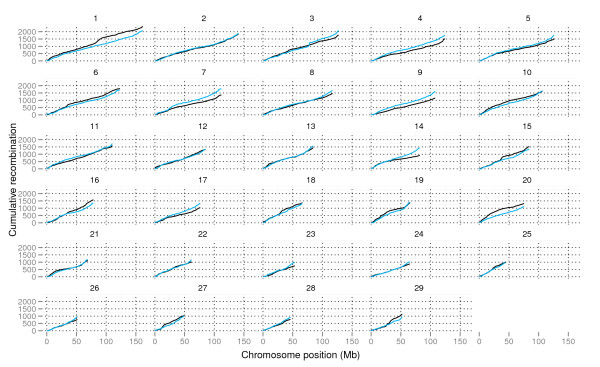
**Recent and historic recombination rate**. Cumulative values of recent recombination rate (blue) and historic recombination rate (black) across each bovine autosomal chromosome is plotted against physical chromosome position (Mb). Chromosome numbers are indicated above each plot.

To investigate recombination patterns across genomic regions, maps describing historic LD levels were constructed for each chromosome based on population recombination rate in the NRF data. By the method presented by Auton and McVean [25], estimates of scaled population recombination rate (ρ = 4cN_e_) [9] were found for each interval between adjacent SNPs for all 29 BTAs with the LDhat software [26]. Following Pritchard and Przeworski [8], historical scaled recombination rate (ρ(h)) was compared with recent scaled recombination rate (ρ(r)) calculated from the genetic map by plotting their cumulative values against physical position (Figure 3).

Correlation between total cumulative ρ(h) and ρ(r) over all chromosomes was found to be 0.84. A reduced ρ(r) relative to ρ(h) for a genomic region could be an indication that animals in the observed pedigree have been under strong artificial selection for traits affected by polymorphisms in that particular region. Regions where ρ(r) was most strikingly reduced relative to ρ(h) were in the middle of BTA1 and in the middle of BTA20. Reduced ρ(r) relative to ρ(h) was also found on BTAs 6, 10,15, 16, 18, 19, 27 and 29, while elevated ρ(r) relative to ρ(h) was found on BTAs 3, 4, 7, 9, 11, 14, and 17.

On BTA1 several QTLs affecting milk production traits have been reported [27-32], and a meta-analysis reported by Khatkar *et al. *[33] indicated presence of three QTLs for milk yield on this chromosome. The BTA20 region centres around a mutation reported to affect protein percentage in the *GHR *gene [34]. Hayes *et al. *[35] reported evidence for strong selection in this region in a study of divergence between dairy cattle and beef cattle. On BTA6 two QTLs affecting milk production traits have been reported in NRF [36,37] and signatures of strong selection have been detected [38].

Elevated population recombination rate may be due to population expansion or gene conversion, while reduced recombination rate may be due to directional selection, genetic drift, gene flow, population substructure or low effective population size [1]. Regions under strong selection in both historic and recent generations might not show differences between ρ(h) and ρ(r).

**Figure 4:**
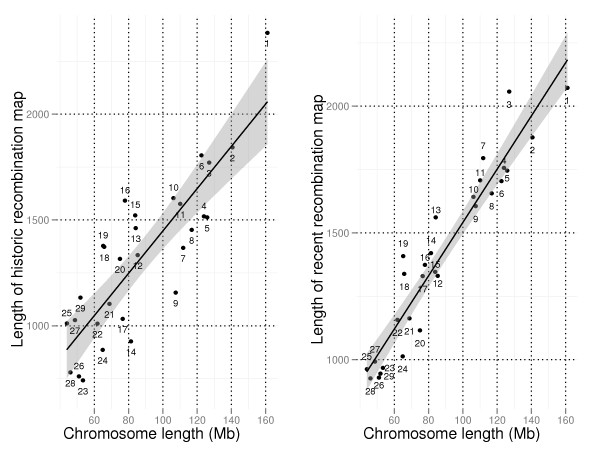
**Recombination maps versus physical chromosome length**. Length of historic recombination map (left) and recent recombination map (right) are plotted against physical chromosome length for each bovine autosomal chromosome. The straight black lines indicate expected recombination map lengths relative to chromosome length and the darker grey regions their 95% confidence intervals.

It was also investigated whether recombination rates per physical distance were related to chromosomal region or chromosome size. Telomeres showed significantly higher values for both recent and historic recombination rate per physical distance than the genome average (p-values 5.72e-12 and 2.97e-8). A negative correlation between recombination rate and chromosome length is expected [10,39-41], and for ρ(h) and ρ(r) correlations of -0.6 and -0.83 was found between length of genetic map and physical chromosome length. Identification of chromosomes with unexpectedly elevated or reduced recombination rate could be identified by looking at outliers deviating from the expected linear relationship between recombination rate and chromosome length. In Figure 4 total cumulative values of recombination rate for ρ(h) and ρ(r) are plotted against physical chromosome length for each bovine chromosome. Expected recombination map lengths relative to chromosome lengths with 95% confidence intervals (CIs) are also indicated in the figure.

It can be seen that ρ(h) is elevated for BTAs 1, 13, 15, 16, 18, 19, 20 and 29, while ρ(r) is elevated for BTAs 3, 7, 13, 18 and 19. Further, reduced ρ(h)is observed for BTAs 4, 5, 7, 8, 9, 14, 17, 23, 24 and 26, while ρ(r)is reduced for BTAs 20 and 24. Consistently elevated ρ(h) and ρ(r) are found for BTAs 13, 18 and 19 and consistently reduced ρ(h) and ρ(r) are found for BTA24. In accordance with results described above, ρ(r) is significantly reduced relative to ρ(h) for BTA1, BTA6 and BTA20.

In addition to chromosome region and chromosome size, sex-specific differences in recombination rates have been reported [42-44]. In this study male genetic maps for NRF were used, which might differ from female genetic maps. The patterns of recombination described here might also be sex-specific.

Moreover, rate of recombination is not uniform across a chromosomal segment and recombination events tend to occur in recombination hotspots [2,3]. It has been estimated that these hotspots occur on average every 50 - 100 kb in the human genome [45,46]. Although the procedure applied here for estimation of ρ(h) incorporates a model accounting for variable recombination rates across chromosomes [26], a SNP density higher than obtained in this study would be required in order to detect such fine-scale recombination hotspots in the bovine genome.

### Quality assessment of Btau_4.0

Approximately 5% of the genomic sequence is expected to be missing in Btau_4.0 [19], and positioning of previously un-positioned contigs could aid completion of the assembly by pointing towards regions of special interest for re-sequencing efforts. Here a comparative analysis of the Btau_4.0 assembly with the human genome Build 19 allowed 4,276 previously un-positioned bovine contigs to be given putative genome positions. Determining recombination events between adjacent markers in an extensive pedigree can be used to construct dense genetic maps, and sufficient information was available from our NRF linkage analysis to validate the positions of 321 of these contigs [19]. Comparative analysis and linkage analysis identified 130 new contig positions as being less than 5 Mb apart (Additional file 4). Even though large synteny blocks exist between species comparative analysis will yield spurious positions. Here 40% of positions identified by comparative analysis were validated by linkage analysis.

**Figure 5:**
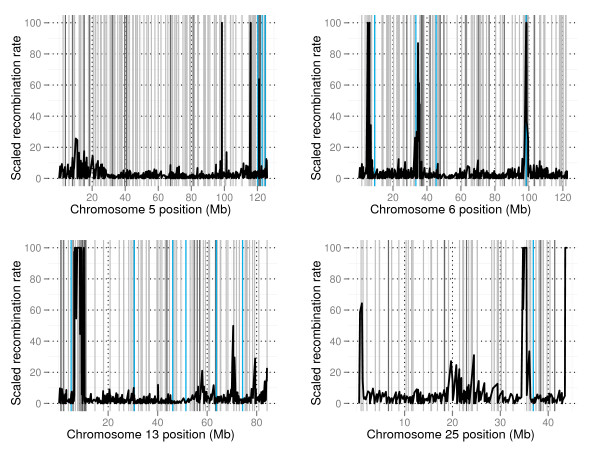
**Quality assessment of the bovine genome assembly**. Scaled recombination rate versus physical distance (kb) is plotted for chromosomes 5 (top left), 6 (top right), 13 (bottom left) and 25 (bottom right). Contig positions predicted by comparative sequence analysis are indicated in light grey and contig positions predicted by linkage analysis are indicated in dark grey. Contigs given similar positions by both methods are indicated in light blue.

To further assess assembly accuracy, population recombination rates between adjacent SNPs were assessed. Regions of putative problematic assembly were identified as extreme values of scaled population recombination rate (ρ) relative to inter-marker distance between adjacent SNP pairs. Extreme values of ρ would be expected for intervals where assembled inter-marker distance was shorter than actual inter-marker distance. In Figure 5 ρ is plotted for BTAs 5, 6, 13 and 25. Contig positions predicted by comparative analysis are indicated in light grey, contig positions predicted by linkage analysis are indicated in dark grey and contig positions validated by similar positions from both comparative analysis and linkage analysis are indicated in light blue. Plots for all chromosomes are given in Additional file 5. From Figure 5 it can be seen that several ρ peaks were located near validated positions for previously un-positioned contigs, which further highlights these regions as erroneous in the original assembly. Examples are the three ρ peak regions on BTA6 around 10 Mb, 35 Mb and 100 Mb, the ρ peak region on BTA5 around 120 Mb, the ρ peak region on BTA13 around 10 Mb and the ρ peak region on BTA25 around 35 Mb. Of the putative contig positions found by comparative analysis 24% lay within 1 Mb of an extreme value for population recombination rate (ρ >10) and of positions found by linkage analysis 27% lay within 1 Mb of an extreme value for population recombination rate. For the 130 contigs given similar positions by comparative analysis and linkage analysis 28% of validated positions lay within 1 Mb of an extreme value for population recombination rate.

Putative contig positions not coinciding with elevated ρ may be due to failure to detect regions with elevated recombination rate, incorrect positioning of un-positioned contigs or un-positioned contigs containing sequence overlap with already assembled contigs. Inability to detect regions with elevated recombination rate could result from surrounding SNPs not containing enough information or from un-positioned contigs being too short to detectably affect ρ. Incorrect positioning of un-positioned contigs could be due to repeat sequence mapping to similar but not equal genomic sequence or random mapping to the wrong position by linkage analysis. Incorrect positioning of un-positioned contigs could also explain why not all of positions found by linkage analysis for the 321 contigs validated positions found by comparative analysis.

An alternative *Bos Taurus *genome assembly (UMD2) was reported by Zimin *et al. *[47]. This assembly had 95% identity with the Btau_4.0 assembly but had more genomic sequence placed on the bovine chromosomes. Regions differing between UMD2 and Btau_4.0 were identified by sequence alignments [47] and some of these regions coincide with regions identified as problematic here. Some examples are the region around 105 Mb on BTA7, the region around 70 Mb on BTA12, the region around 10 Mb on BTA13, the region around 30 Mb on BTA18 and the proximal ends of BTA20, BTA21 and BTA28 (Additional file 5).

Genotyping of SNPs positioned on un-positioned contigs in a large pedigree and consequent linkage analyses as described here provide useful information for improving the current bovine genome assembly (Btau_4.0). The approach will gain even higher power and more accurate predictions as denser genetic maps become available. Likewise comparative sequence analysis would be a good supplement for correct contig or scaffold positioning.

## Conclusions

Low levels of LD were observed in NRF relative to some of the breeds from which this breed originates. This is likely due to elevated heterogeneity in NRF from historic admixture, recent attempts to maintain a large effective population size through control of inbreeding and gene flow through import of sires from other Nordic countries. Reports from breeds other than those assessed in this study have described more rapid decline in r^2 ^at short distances [17,18] than was found in NRF. The results suggested that to obtain an expected r^2 ^between markers and a causal polymorphism of at least 0.5 for genome-wide association studies in NRF, approximately one SNP every 15 kb or a total of 200,000 SNPs would be required for the 2.87 Gb genome.

For well known QTL regions for milk production on BTA1, BTA6 and BTA20, map length based on historic recombination was greater than map length based on recent recombination in NRF. Selective sweeps have previously been identified for the QTL regions on BTA20 [35] and BTA6 [38]. Reduced ρ(r) relative to ρ(h) was also found on BTAs 10,15, 16, 18, 19, 27 and 29, while elevated ρ(r) relative to ρ(h) was found on BTAs 3, 4, 7, 9, 11, 14, and 17.

While over 95% of the total genome sequence is included in bovine genomic assembly Btau_4.0, problematic regions exists and should be identified to facilitate assembly completion. Here such regions were identified by combining comparative sequence analysis, linkage analyses and detection of regions with extreme values of population recombination rate.

## Methods

### Genotyping and initial filtering

The Affymetrix 25 K MIP array [48] was used to genotype 2,589 NRF sires with paternal half-sib pedigree structure. In addition, 53 Holstein, 40 Finnish Ayrshire, 19 Sided Troender and Nordland Cattle and 39 Icelandic sires were genotyped. Genotypes were filtered for discordants (<2.5%), MAF (>0.025) and genotyped percentage (>75%). After initial filtering 17,483 SNPs remained. MAF were calculated for these SNPs with the Haploview 4.1 software [49].

### Genetic map construction

Genetic maps for each of the 29 BTAs were constructed by use of the CRI-MAP 2.4 package [50]. The map file created by use of the CRI-MAP *fixed *option was checked for elevated recombination rates between adjacent SNPs. Elevated recombination rates could be an indication of a wrongly positioned contig in the assembly. SNPs with genetic distance >6 cM between its two flanking SNPs or a genetic distance >4 cM between itself and one of its flanking SNPs were identified as suspicious and temporarily taken out of the genetic map. The CRI-MAP *chrompic *option was used to identify double recombinants. Double recombinants were manually inspected and corrected. SNPs showing up as double recombinants in more than 30 animals from 5 or more families were identified as suspicious and temporarily taken out of the genetic map. The *fixed *and *chrompic *procedures were repeated until no SNPs showed a genetic distance >6 cM between its two flanking SNPs or a genetic distance >4 cM between itself and one of its flanking SNPs. The SNPs temporarily taken out of the genetic map were attempted repositioned by use of the CRI-MAP *twopoint *option. SNPs mapping more strongly to positions within 2.5 Mb of their original positions were not repositioned. The genetic maps has previously been reported in Liu *et al. *[19].

### Haplotypes and missing genotypes

The PHASE software [51] and the locally developed CRIHAP package were applied to utilize both linkage and LD information for determining haplotypes and impute missing genotypes.

### Principal component analysis

A principal component analysis of the genomic relationship matrix among individuals of different and the same breed [23] was conducted to evaluate genetic distances between breeds.

### Recombination rate distribution

Recombination rates in telomeric regions were compared to average recombination rates across chromosomes. Mean recombination rate per bp in a 10 Mb telomeric region for all autosomes was compared to overall mean recombination rate per bp. A t-test was used to compare means.

### Linkage disequilibrium

Estimates of pair-wise linkage disequilibrium measure r^2 ^were calculated with the Haploview 4.1 software [49].

### Population recombination rate

Scaled population recombination rate (ρ) between adjacent markers relative to inter marker distances was estimated using the LDhat 2.1 software [26] with haplotypes from 17,347 SNPs distributed on the 29 BTAs. The LDhat 2.1 software incorporates a model which allows for variable recombination rates across chromosomes [26]. The reversible-jump markov chain monte carlo (rjMCMC) chain was run for 10,000,000 iterations, performing 5000 iterations between each sample. A block penalty of 5 was applied and the first 500,000 iterations were discarded as burn-ins. Some extreme values of ρ were observed, which could to be due to wrongly assembled contigs or other assembly artefacts. To determine historical recombination rate (ρ(h)) extreme values were corrected by replacing extreme values of ρ by a maximum value (max interval ρ = 10). Less than 5% of intervals between adjacent SNPs had ρ higher than this maximum value. To determine values of recent scaled population recombination rate from the observed pedigree (ρ(r)), pedigree based estimates of recombination (c) was scaled by a factor corresponding to 4N_e _(from ρ = 4cN_e_) [3,8]. The applied scaling factor ρ(h)/c = 4N_e _was found by taking average values of total cumulative ρ(h) and total cumulative c for all autosomes. Cumulative values of recombination over each interval was used because we were interested in comparing a recent population recombination map based on the observed pedigree with a historic population recombination map, rather than comparing point estimates of recombination rate per distance. Cumulative interval values for ρ(r) and ρ(h) were found by multiplying recombination rate per length unit for each interval with interval length and then calculating cumulative values across each autosomal chromosome.

### Positioning previously un-positioned contigs

Positioning of previously un-positioned contigs from the bovine genome sequencing (Btau_4.0) [19] was done both by comparative sequence analysis with the human genome and by linkage analysis with already positioned SNPs in the bovine genome (Btau_4.0). In the comparative sequence analysis positioning of un-positioned bovine contigs was performed by combining MegaBLAST [52] searches for un-positioned contigs against the human genome Build 19 followed by MegaBLAST searches of hits in the human genome against the bovine genome (Btau_4.0). The first search revealed which areas of the human genome was most similar to each unknown contig, while the second search mapped those areas in the human genome sequence back to the bovine genome sequence. When two human sequences from one chromosomal region gave MegaBLAST hits against two sequences of a bovine chromosomal region it was assumed that the sequence between those human sequences on the human chromosome would also have similarity to the bovine genomic sequence between the two hits on the bovine chromosome. Bovine positions were predicted for 4,276 previously un-positioned contigs by this comparative method. Moreover, linkage analysis was conducted to position 568 SNPs distributed on 321 of the un-positioned contigs. The *twopoint *option in CRI-MAP 2.4 [50] was used to map these 568 SNPs to SNPs already positioned in the bovine genome assembly. The results from linkage analysis were also presented in Liu *et al. *[19].

## Authors' contributions

MS construction of genetic maps, linkage analyses, linkage disequilibrium analyses for Norwegian Red cattle, recombination rate analyses and writing of manuscript. MK molecular genetic studies and initial data cleaning. BJH principal component analysis, linkage disequilbrium analysis for breed comparison and assistance in drafting the manuscript. HG comparative sequence analysis. SL initial data cleaning, construction of genetic maps, conceived of the study, coordination and assistance in drafting the manuscript. All authors helped finalize the manuscript and read and approved of the final version.

## Supplementary Material

Additional file 1 **Inter-marker distance distribution**. Genome-wide distribution of distance (bp) between adjacent SNPs. Click here for file

Additional file 2 **Minor allele frequency**. Genome-wide distribution of minor allele frequencies after filtering (>0.025). Click here for file

Additional file 3 **Chromosomal linkage disequilibrium**. Number of SNPs, number of SNP pairs, mean chromosomal r^2 ^and mean r^2 ^for inter-marker distances <10 Mb for the 29 BTAs. Click here for file

Additional file 4 **Positioning unpositioned contigs**. Comparative sequence analysis (CSA) contig positions were compared with the positions predicted by linkage analysis (LA) presented in Liu *et al. *[19]. The table shows 130 contigs unpositioned in the genome assembly (Btau_4.0) for which contig positions from these two prediction methods are less than 5 Mb apart. Contig, BTA, position given by CSA and position given by LA is presented. Click here for file

Additional file 5 **Quality assessment of the bovine genome assembly Btau_4.0**. Scaled recombination rate versus physical distance (kb) is plotted for all 29 autosomal bovine chromosomes. Contig positions predicted by comparative sequence analysis are indicated in light grey and contig positions predicted by linkage analysis are indicated in dark grey. Contigs given similar positions by both methods are indicated in light blue. Click here for file
