# Supplementary material for: Recent and historical recombination in the admixed Norwegian Red cattle breed
Source: BMC Genomics. 2011 Jan 14;12:33. doi: 10.1186/1471-2164-12-33 (PMC3030550; doi:10.1186/1471-2164-12-33)

Additional file 1- Inter-marker distance distribution

Figure A1- Inter-marker distance distribution

Genome-wide distribution of distance (bp) between adjacent SNPs.

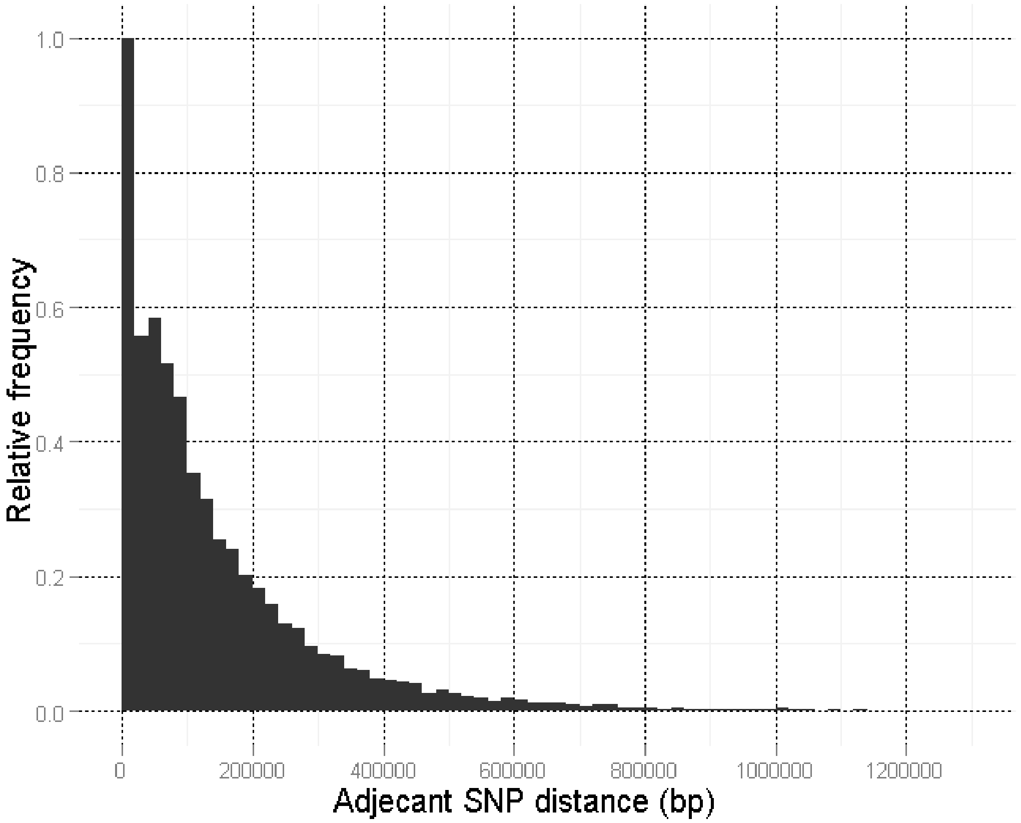

Supplement: Additional file 1 — Inter-marker distance distribution. Genome-wide distribution of distance (bp) between adjacent SNPs. [file 1471-2164-12-33-S1.PDF]
