# Supplementary material for: Recent and historical recombination in the admixed Norwegian Red cattle breed
Source: BMC Genomics. 2011 Jan 14;12:33. doi: 10.1186/1471-2164-12-33 (PMC3030550; doi:10.1186/1471-2164-12-33)

Additional file 2 – Minor allele frequency

Figure A2 – Minor allele frequency

Genome-wide distribution of minor allele frequencies after filtering (>0.025).

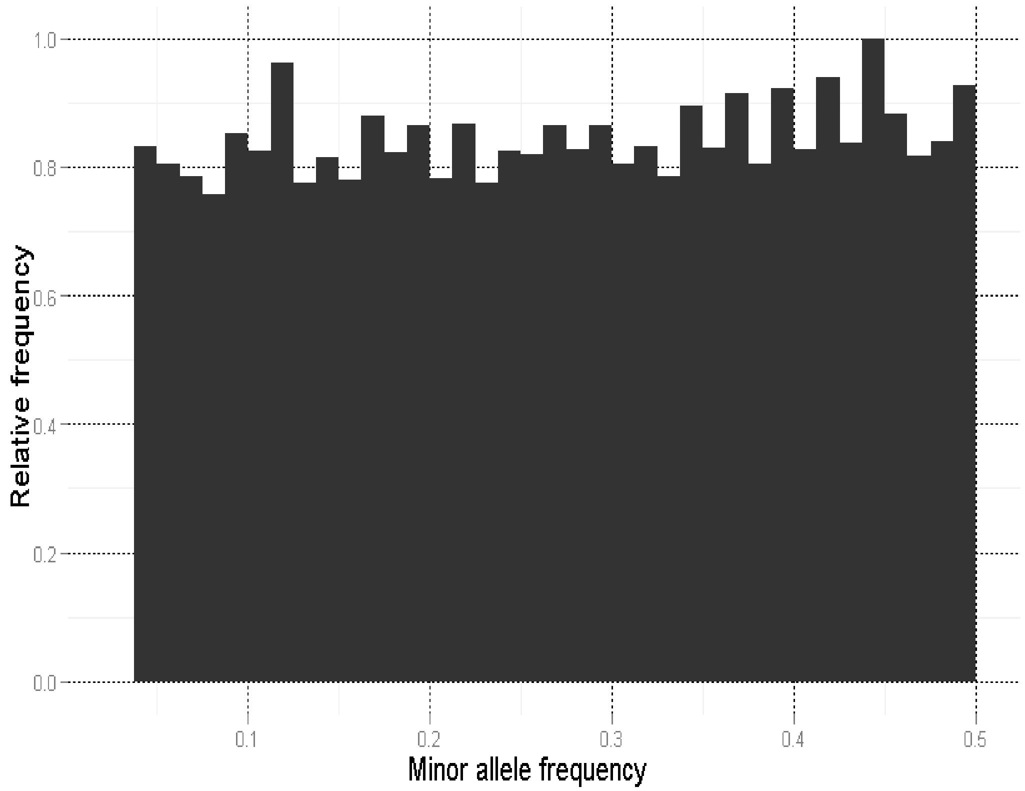

Supplement: Additional file 2 — Minor allele frequency. Genome-wide distribution of minor allele frequencies after filtering (>0.025). [file 1471-2164-12-33-S2.PDF]
