# Supplementary material for: Recent and historical recombination in the admixed Norwegian Red cattle breed
Source: BMC Genomics. 2011 Jan 14;12:33. doi: 10.1186/1471-2164-12-33 (PMC3030550; doi:10.1186/1471-2164-12-33)
Supplement: Additional file 3 — Chromosomal linkage disequilibrium. Number of SNPs, number of SNP pairs, mean chromosomal r2 and mean r2 for inter-marker distances <10 Mb for the 29 BTAs. [file 1471-2164-12-33-S3.PDF]

Additional file 3 – Chromosomal linkage disequilibrium

Table A3 – Chromosomal linkage disequilibrium

Number of SNPs, number of SNP pairs, mean chromosomal r^2 and mean r^2 for inter-marker distances <10Mb for the 29 BTAs.

BTA	Number of SNPs	Pairs	r^2	$SD(r^2)$	$r^2_{0\text{-}10Mb}$	$SD(r^2_{0\text{-}10Mb})$
1	1105	582660	0.00838	0.02921	0.0357	0.07459
2	972	445096	0.00988	0.03332	0.0359	0.07899
3	855	337431	0.00949	0.03248	0.03505	0.07482
4	871	350703	0.01107	0.03744	0.04255	0.08482
5	775	271216	0.01243	0.03844	0.04397	0.08511
6	859	355746	0.01055	0.03523	0.03835	0.07904
7	704	232903	0.01009	0.03551	0.03514	0.07779
8	788	290703	0.012	0.03595	0.04035	0.07735
9	676	215496	0.01207	0.03811	0.03871	0.08003
10	718	243951	0.01069	0.03705	0.03714	0.08009
11	776	285390	0.01003	0.03258	0.0344	0.07056
12	618	182710	0.01212	0.03866	0.03553	0.07509
13	610	172578	0.01219	0.04267	0.03735	0.08337
14	583	159330	0.01422	0.04577	0.04072	0.08693
15	534	131328	0.01373	0.04425	0.0385	0.08447
16	549	143380	0.01158	0.03989	0.03092	0.07479
17	566	150975	0.0139	0.04143	0.03636	0.07573
18	457	97903	0.01459	0.04818	0.03532	0.0829
19	439	87571	0.01131	0.0375	0.02773	0.06492
20	563	147696	0.01393	0.04265	0.03619	0.07924
21	420	81810	0.01564	0.04146	0.03639	0.07027
22	446	94395	0.01634	0.04917	0.03558	0.08181
23	379	67896	0.01609	0.04696	0.03557	0.07478
24	466	97020	0.01317	0.03624	0.03033	0.06263
25	287	39621	0.01552	0.04192	0.02948	0.06131
26	361	59685	0.01521	0.04719	0.0326	0.07409
27	332	52650	0.01517	0.04653	0.03085	0.07187
28	317	48205	0.01423	0.0424	0.02824	0.06491
29	321	47586	0.01567	0.04818	0.03273	0.07685
all	17357	4890974	0.01188	0.03854	0.03625	0.07768
