# Supplementary material for: Recent and historical recombination in the admixed Norwegian Red cattle breed
Source: BMC Genomics. 2011 Jan 14;12:33. doi: 10.1186/1471-2164-12-33 (PMC3030550; doi:10.1186/1471-2164-12-33)
Supplement: Additional file 4 — Positioning unpositioned contigs. Comparative sequence analysis (CSA) contig positions were compared with the positions predicted by linkage analysis (LA) presented in Liu et al. [19]. The table shows 130 contigs unpositioned in the genome assembly (Btau_4.0) for which contig positions from these two prediction methods are less than 5 Mb apart. Contig, BTA, position given by CSA and position given by LA is presented. [file 1471-2164-12-33-S4.PDF]

Additional file 4 – Positioning unpositioned contigs

Table A4 - Positioning unpositioned contigs

Comparative sequence analysis (CSA) contig positions were compared with the positions predicted by linkage analysis (LA) presented in Liu *et al*. [19]. The table shows 130 contigs unpositioned in the genome assembly (Btau_4.0) for which contig positions from these two prediction methods are less than 5Mb apart. Contig, BTA, position given by CSA and position given by LA is presented.

Contig	BTA	CSA pos (bp)	LA pos (bp)
ChrUn.004.768	1	26281956	28879298
ChrUn.004.85	1	50891360	47153415
ChrUn.004.1001	1	61874192	65957389
ChrUn.004.1144	1	74227536	74099262
ChrUn.004.371	1	84130992	83842131
ChrUn.004.321	1	95731182	99481432
ChrUn.004.11	1	107740683	103459113
ChrUn.004.1062	1	115643895	115969634
ChrUn.004.29	1	117040459	116820290
ChrUn.004.747	1	117040459	116934834
ChrUn.004.766	2	36098554	36426908
ChrUn.004.765	2	41317799	40975539
ChrUn.004.297	2	61251434	58063626
ChrUn.004.1449	2	64218868	65535887
ChrUn.004.3074	2	88458990	92842235
ChrUn.004.382	2	90856067	93320532
ChrUn.004.1701	2	111956989	116100367
ChrUn.004.404	2	120789554	120578545
ChrUn.004.1480	3	66838524	69718616
ChrUn.004.3871	3	79358875	76728899
ChrUn.004.5148	3	79358875	76997488
ChrUn.004.25	3	125654871	124219358
ChrUn.004.3881	4	21574657	21635538
ChrUn.004.761	4	33910203	35636863
ChrUn.004.982	4	44630601	41683742
ChrUn.004.700	4	61880729	58473419
ChrUn.004.712	4	100751169	99881861
ChrUn.004.2120	4	119106981	121214561
ChrUn.004.46	5	120128110	120200555
ChrUn.004.101	5	124029455	122807308
ChrUn.004.152	5	124029455	124690330
ChrUn.004.1978	6	7421145	9256262
ChrUn.004.14	6	36811562	33498581
ChrUn.004.688	6	47739165	45406895
ChrUn.004.181	6	98157363	98421268
ChrUn.004.2758	7	44286170	43289003
ChrUn.004.7	7	58031854	56735107
ChrUn.004.538	7	83240720	83283438
ChrUn.004.47	7	111786467	109447065
ChrUn.004.2458	8	22646403	22996780
ChrUn.004.1528	8	77992562	77818564
ChrUn.004.1112	8	85426569	85064076
ChrUn.004.1769	8	96160463	97868552
ChrUn.004.1350	8	105033113	107991032
ChrUn.004.310	9	59473956	56206860
ChrUn.004.22	9	63692465	62848329
ChrUn.004.4916	9	98928035	99397801
ChrUn.004.1768	9	99823180	101723746
ChrUn.004.360	10	293535	234438
ChrUn.004.3037	10	50630272	49803491
ChrUn.004.519	10	52653988	51216078
ChrUn.004.1250	10	83209908	79864903
ChrUn.004.114	10	95632496	95372032
ChrUn.004.1844	10	99977821	103295043
ChrUn.004.1919	11	2441949	3612595
ChrUn.004.705	11	3435759	4838605
ChrUn.004.704	11	12876305	12891182
ChrUn.004.475	11	65530279	65893071
ChrUn.004.4462	11	90267750	89018683
ChrUn.004.135	11	109420358	110120453
ChrUn.004.4	12	3622333	219151
ChrUn.004.2425	12	25695359	29298576
ChrUn.004.1654	12	36223108	35745307
ChrUn.004.23	12	55825173	57534765
ChrUn.004.51	12	60698388	59088156
ChrUn.004.8	13	8129958	5056004
ChrUn.004.3218	13	30096787	30551956
ChrUn.004.3124	13	47000000	46102567

Contig	BTA	CSA pos (bp)	LA pos (bp)	Contig	BTA	CSA pos (bp)	LA pos (bp)
ChrUn.004.256	13	53922982	51389627	ChrUn.004.1047	20	21515643	19217974
ChrUn.004.288	13	64065293	63708121	ChrUn.004.1321	20	39352014	36458435
ChrUn.004.1967	13	73027223	74341206	ChrUn.004.374	20	59872532	60203096
ChrUn.004.3650	13	75876281	74341206	ChrUn.004.816	21	16878708	21334087
ChrUn.004.209	14	1888669	1647400	ChrUn.004.673	21	34453544	30105866
ChrUn.004.1	14	35153552	30580221	ChrUn.004.909	21	52240388	50972300
ChrUn.004.402	14	49666812	47064609	ChrUn.004.582	21	59001500	61410494
ChrUn.004.2216	14	52288903	49652936	ChrUn.004.2235	21	60354669	61410494
ChrUn.004.2679	14	70992550	70750938	ChrUn.004.177	21	68643427	64050679
ChrUn.004.1073	15	7126997	4707548	ChrUn.004.201	21	68830449	67177207
ChrUn.004.423	15	6467493	5727843	ChrUn.004.886	22	23071612	23043793
ChrUn.004.28	15	7126997	10819187	ChrUn.004.187	22	50136329	51965281
ChrUn.004.289	15	44694489	42584302	ChrUn.004.340	23	48042797	46627945
ChrUn.004.1936	15	58220038	59000656	ChrUn.004.1225	23	52000000	47444533
ChrUn.004.2	16	20886394	18185763	ChrUn.004.3014	24	38715301	38708428
ChrUn.004.3	16	34310295	30983359	ChrUn.004.894	24	49263691	50125408
ChrUn.004.721	16	32323091	33295121	ChrUn.004.4000	24	55804015	58376977
ChrUn.004.481	16	77473196	75179404	ChrUn.004.242	25	38641412	36887600
ChrUn.004.108	16	76018320	77581778	ChrUn.004.5408	25	38039057	36887600
ChrUn.004.492	17	55350140	54752610	ChrUn.004.1167	26	23996164	23755539
ChrUn.004.226	17	61858842	57827509	ChrUn.004.377	26	46864751	47322086
ChrUn.004.428	17	69725288	71543454	ChrUn.004.240	26	50194359	49465313
ChrUn.004.1536	17	68540063	72151921	ChrUn.004.2193	26	51734544	51054953
ChrUn.004.794	18	10433035	10168415	ChrUn.004.276	28	298870	3035821
ChrUn.004.660	18	15139873	16858980	ChrUn.004.5489	28	2492789	3220800
ChrUn.004.354	18	26457691	23383717	ChrUn.004.261	28	510799	3460323
ChrUn.004.2172	19	41115255	42058156	ChrUn.004.432	29	33931870	34967060
ChrUn.004.706	19	53560899	54452705	ChrUn.004.65	29	47533875	46824437
ChrUn.004.241	19	60822297	58035657	ChrUn.004.171	29	51580000	47525062
ChrUn.004.331	19	63091394	61096438	ChrUn.004.137	29	51580000	51539390
ChrUn.004.1236	20	4577209	4745147	ChrUn.004.163	29	51580000	51539390
