# Supplementary material for: Recent and historical recombination in the admixed Norwegian Red cattle breed
Source: BMC Genomics. 2011 Jan 14;12:33. doi: 10.1186/1471-2164-12-33 (PMC3030550; doi:10.1186/1471-2164-12-33)
Supplement: Additional file 5 — Quality assessment of the bovine genome assembly Btau_4.0. Scaled recombination rate versus physical distance (kb) is plotted for all 29 autosomal bovine chromosomes. Contig positions predicted by comparative sequence analysis are indicated in light grey and contig positions predicted by linkage analysis are indicated in dark grey. Contigs given similar positions by both methods are indicated in light blue. [file 1471-2164-12-33-S5.PDF]

Additional file 5 - Quality assessment of the bovine genome assembly Btau_4.0

Figure A5 - Quality assessment of the bovine genome assembly Btau_4.0
Scaled recombination rate versus physical distance (kb) is plotted for all 29 autosomal bovine chromosomes. Contig positions predicted by comparative sequence analysis are indicated in light grey and contig positions predicted by linkage analysis are indicated in dark grey. Contigs given similar positions by both methods are indicated in light blue.

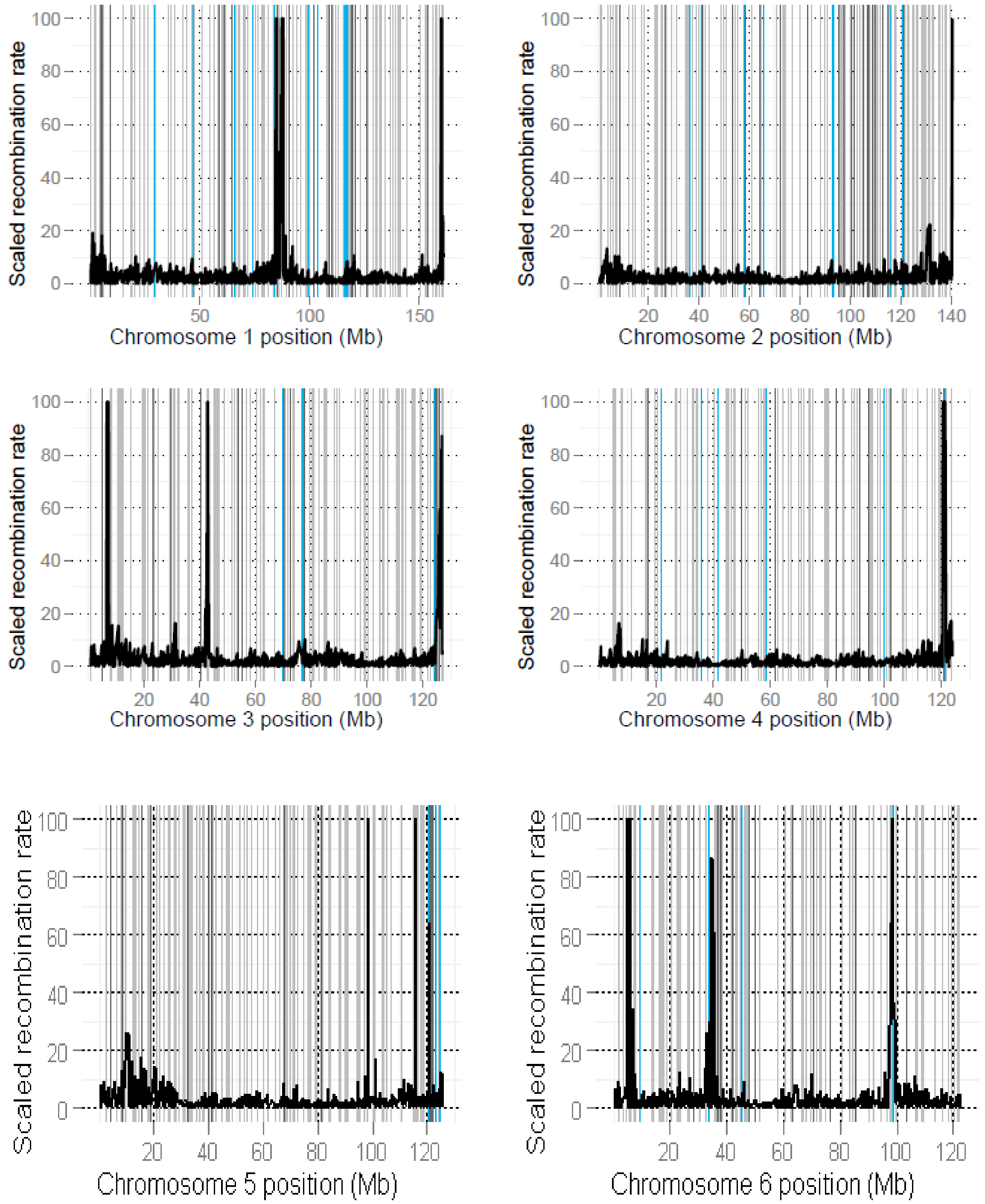

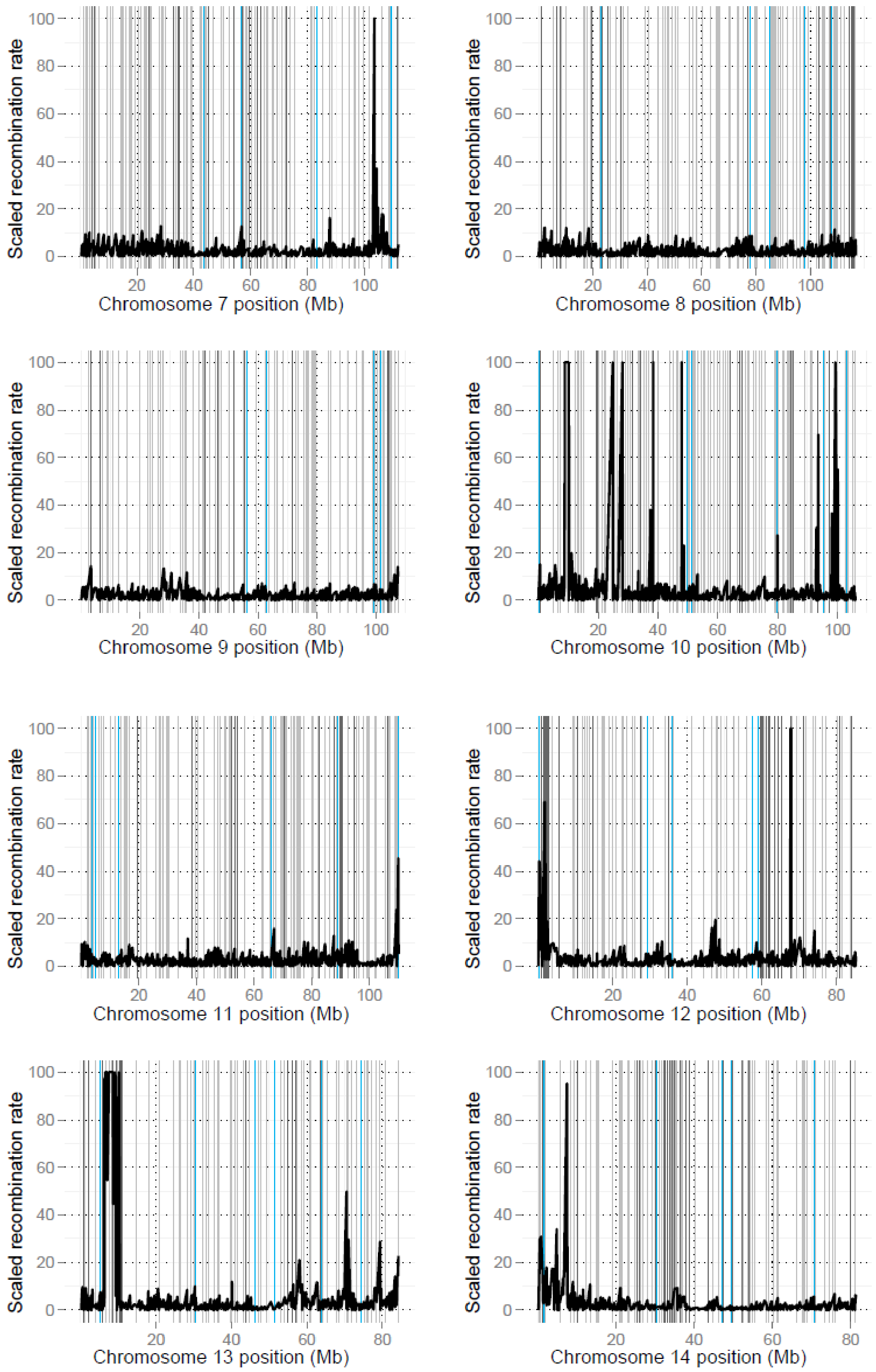
Scaled recombination rate
100
80
60
40
20
0
20
40
60
80
100
Chromosome 7 position (Mb)
Chromosome 8 position (Mb)
Chromosome 9 position (Mb)
Chromosome 10 position (Mb)
Chromosome 11 position (Mb)
Chromosome 12 position (Mb)
Chromosome 13 position (Mb)
Chromosome 14 position (Mb)

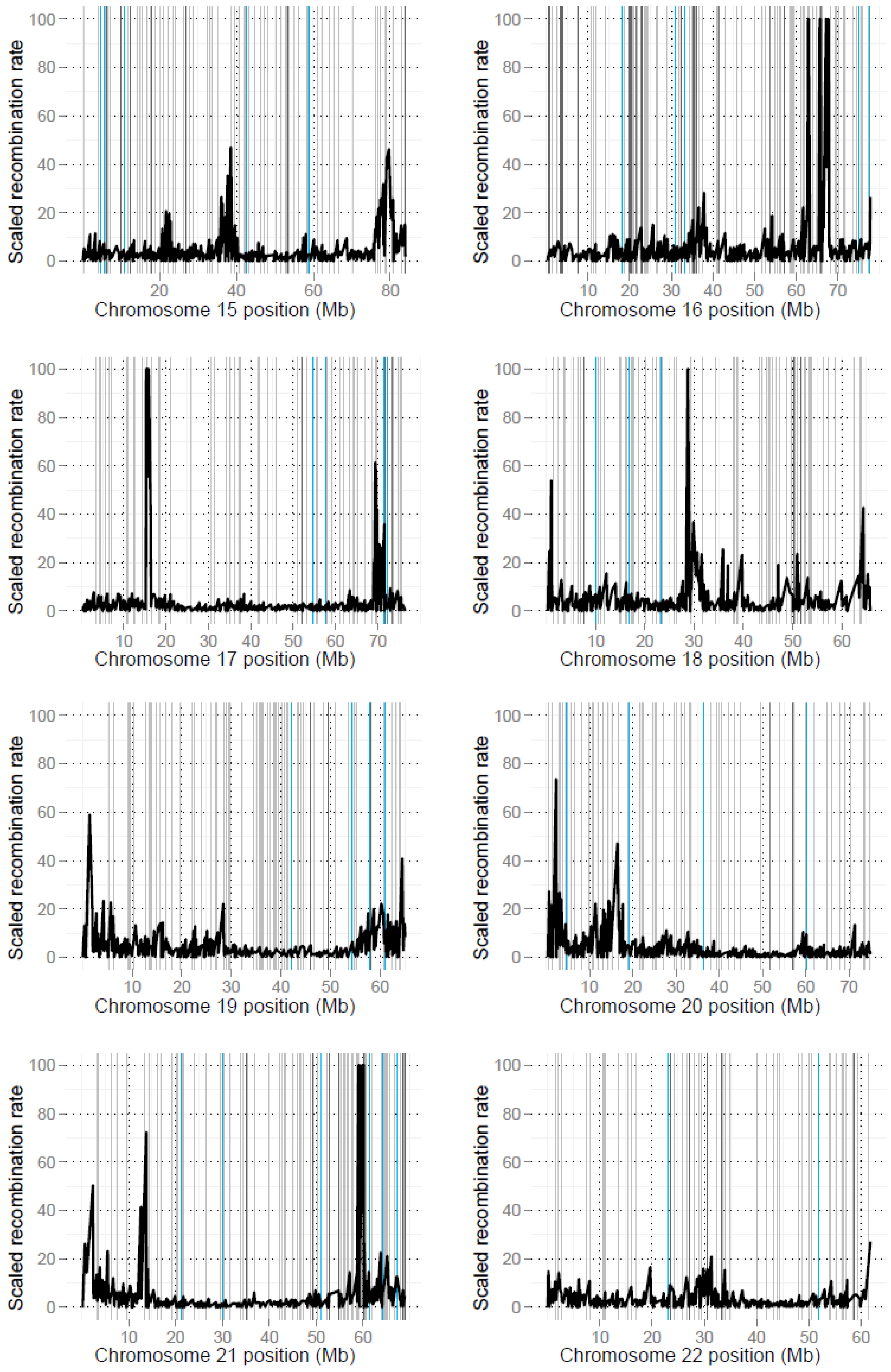
Scaled recombination rate
Chromosome 15 position (Mb)
Chromosome 16 position (Mb)
Chromosome 17 position (Mb)
Chromosome 18 position (Mb)
Chromosome 19 position (Mb)
Chromosome 20 position (Mb)
Chromosome 21 position (Mb)
Chromosome 22 position (Mb)

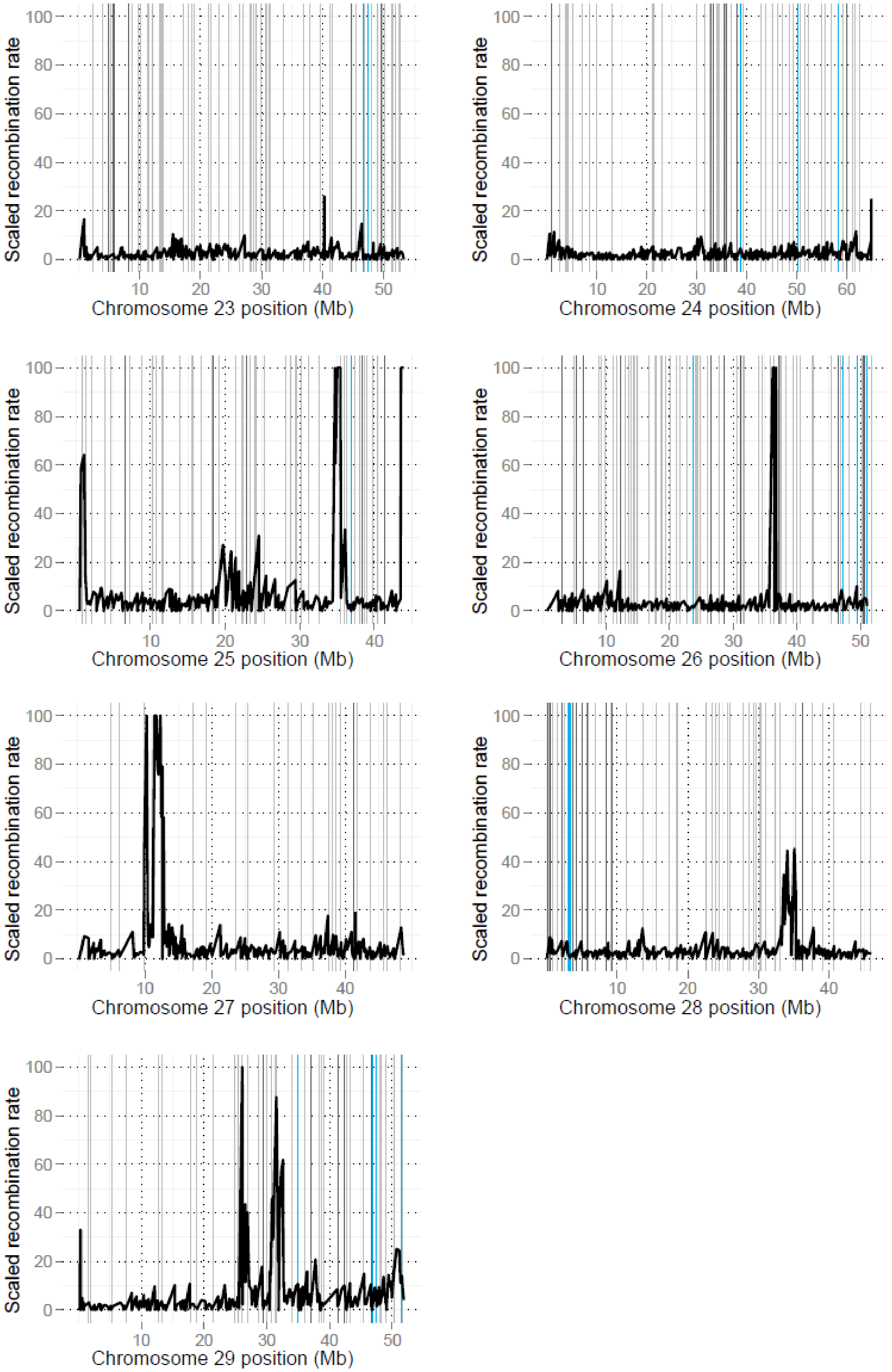
Scaled recombination rate
100
80
60
40
20
0
10
20
30
40
50
60
Chromosome 23 position (Mb)
Chromosome 24 position (Mb)
Chromosome 25 position (Mb)
Chromosome 26 position (Mb)
Chromosome 27 position (Mb)
Chromosome 28 position (Mb)
Chromosome 29 position (Mb)
